# Emergency, critical and operative care services for effective primary care

**DOI:** 10.2471/BLT.20.280016

**Published:** 2020-11-01

**Authors:** Teri A Reynolds, Ann-Lise Guisset, Suraya Dalil, Pryanka Relan, Shannon Barkley, Edward Kelley

**Affiliations:** a Integrated Health Services, World Health Organization, Avenue Appia 20, 1211 Geneva 27, Switzerland.; b Special Programme on Primary Health Care, World Health Organization, Geneva, Switzerland.

A primary health-care approach to service delivery places primary care at the core of integrated health services, ensuring that systems are responsive to people’s needs, values and preferences. At its best, primary care directly addresses most of the health care needs of most people, while also providing early recognition of dangerous conditions, timely resuscitation and targeted referral when needed. Shortly after the Declaration of Alma-Ata, World Health Organization (WHO) Director-General Halfdan Mahler said: “A health system based on primary care cannot be realized without support from a network of hospitals,”^1^ establishing a vision of integrated service delivery that was reaffirmed in the Declaration of Astana. Hospital-dominated health-system planning, however, has been consistently identified as an important obstacle to fully realized primary health care. The 2008 World Health Report specifies that “health systems do not spontaneously gravitate towards primary health-care values, in part because of a disproportionate [...] hospital-centrism.”^2^

The strategic and operational transformations associated with primary health care, however, need not entail an opposition between preventive–promotive approaches and acute or advanced treatments. This opposition is a false dichotomy that undermines the potential for real service integration: both primary care and hospital platforms support prevention and treatment, and hospitals are increasingly recognized as an essential part of the solution rather than a problem.^3^

A primary health-care approach to service delivery optimizes both primary and hospital-based care by taking a population-based planning approach that accounts for the distinct and complementary capacities of these platforms, as well as their profound interdependence. Effective health systems must respond to people’s needs for time-sensitive and out-of-hours emergency care and ensure access to critical care and operative services that can only be delivered safely in a hospital setting. Integrated people-centred service delivery requires emergency, critical and operative care (ECO) services that are linked to communities through primary care and by communication, transportation, referral and counter-referral mechanisms. We use the term ECO-system to refer to these emergency, critical and operative care services and the mechanisms that ensure they are accessible to the people who need them. Only integrated planning that places longitudinal primary care relationships at the centre of this ECO-system will ensure timely and appropriate access to needed care across the life course.

Similar to primary-care clinics, prehospital and emergency unit services sit at the interface of the community and the hospital, providing first-contact care. While the course of a chronic illness may be punctuated by severe exacerbations that can only be treated with emergency care, the implications of these acute episodes can only be fully understood and their recurrence prevented in the context of longitudinal primary care.^4^ An acute exacerbation of asthma or heart failure, for example, may entail a change in a chronic treatment regimen or in baseline diagnostic classification. Even with optimal preventive care, a range of conditions may require operative care across the life course, including for acute injury, safe childbirth and a range of communicable and noncommunicable diseases. The global need for surgical and anaesthesia services greatly outstrips their availability,^5^ and the capacity of primary care to provide timely, accurate and appropriate referral is essential to optimal use of this scarce resource.

Never has the interdependence of these platforms been as evident as in the current pandemic context, with the simultaneous need for safe access to health services close to home, early recognition and intervention to pre-empt severe illness, and timely targeted referral and counter-referral to optimize use of extremely scarce resources. Responding effectively to the coronavirus disease 2019 while maintaining essential health service delivery will require primary health-care approaches that include robust ECO-systems.

Countries and regions continue to emphasize the importance of timely access to emergency, critical and operative services,^6^^–^^8^ and they increasingly highlight the need for integrated approaches to service organization.^9^^–^^12^ In response to these calls, as part of its recent transformation process, WHO has established a dedicated Clinical Services and Systems unit in the Department of Integrated Health Services. This unit brings together for the first time the organization’s work on integrated clinical delivery channels, including primary care, emergency care, critical care and surgery and anaesthesia care, with a new focus on people’s movement across the health system. The mandate of this unit is complemented by a special initiative on health services planning, organization and management designed to support district and other local health authorities and managers to translate guidance into action. Through these and other efforts across the levels of the organization, WHO is committed to supporting countries’ efforts to strengthen health service delivery through enabling ECO-systems built around robust primary care.
